# Association of intraocular pressure and postoperative nausea and vomiting after microvascular decompression - a prospective cohort study

**DOI:** 10.1186/s12871-022-01665-x

**Published:** 2022-04-30

**Authors:** Yuantao Hou, Hansheng Liang, Cungang Fan, Ruen Liu, Yi Feng

**Affiliations:** 1 Department of Anesthesiology, Peking University People’s Hospital, No. 11 Xizhimen South Street, Xicheng District, Beijing, China; 2 Department of Neurosurgery, Peking University People’s Hospital, No. 11 Xizhimen South Street, Xicheng District, Beijing, China

**Keywords:** Hemifacial spasm, Intraocular pressure, Microvascular decompression, Nausea, Vomiting

## Abstract

**Background:**

Postoperative nausea and vomiting is common in patients receiving microvascular decompression. In the current study, we examined whether postoperative nausea and vomiting is associated with reduced intraocular pressure (IOP) after microvascular decompression, a measure that reflects intracranial pressure.

**Methods:**

This is a prospective cohort study. Adult patients scheduled for microvascular decompression surgery for hemifacial spasm between January 2020 and August 2020 were eligible. IOP was measured immediately before anesthesia induction and 30 min after patients regained complete consciousness using non-contact tonometry. IOP reduction was defined by at least 1 mmHg decrease vs. preoperative baseline. The primary outcome was vomiting on postoperative day 1.

**Results:**

A total of 103 subjects were enrolled. IOP was reduced in 56 (54.4%) subjects. A significantly greater proportion of patients with IOP reduction had vomiting on postoperative day 1 (51.8% (29/56) vs. 23.4% (11/47) in those without IOP reduction; *p* = 0.003). In the multivariate regression analysis, vomiting on postoperative day 1 was associated with female sex [odds ratio = 7.87, 95% CI: 2.35–26.32, *p* = 0.001] and IOP reduction [odds ratio = 2.93, 95% CI: 1.13–7.58, *p* = 0.027].

**Conclusions:**

In patients undergoing microvascular decompression surgery, postoperative IOP reduction is associated with postoperative vomiting.

**Trial registration:**

Chinese Clinical Trial Registry: ChiCTR2000029083. Registered 13 January 2020.

**Supplementary information:**

The online version contains supplementary material available at 10.1186/s12871-022-01665-x.

## Background

Microvascular decompression (MVD) is the standard treatment for hyperactive dysfunctional cranial nerve syndromes (such as trigeminal neuralgia, hemifacial spasm, and glossopharyngeal neuralgia) in patients who do not respond to or tolerate pharmacological treatments [1, 2]. A significant proportion of patients experience severe postoperative nausea and vomiting (PONV) in the first 24 h after surgery [3–5]. PONV increases complications (e.g., surgical site bleeding, electrolyte disturbance, dehydration, and aspiration) and delays postoperative recovery [2, 6].

PONV has been partly attributed to loss of cerebrospinal fluid (CSF) and sudden reduction of intracranial pressure (ICP) [2]. This circumstance is similar to the post-dural puncture headache, in which CSF loses rapidly, and is often accompanied by nausea and vomiting [7]. Treatments for PONV, including prone position, rehydration, and autologous epidural blood patch, are based on restoring ICP [8].

ICP is often determined via lumbar puncture, an invasive procedure with a risk of nerve injury and infection. Intraocular pressure (IOP) is widely used as a surrogate monitoring [9]. Compared with other methods, IOP monitoring is easy, fast, and inexpensive. We conducted a prospective study to examine PONV in patients undergoing MVD surgery using air puff tonometry. The rate of PONV was compared between patients with significant IOP reduction (at least 1 mmHg decrease from the pre-operative baseline) vs. those without IOP reduction after MVD surgery.

## Methods

### Ethics, consent and permission

 This study was approved by the Ethics Committee of Peking University People’s Hospital (#2019PHB271-01; December 31th 2019). Written informed consents were obtained from all participants. The trial was registered at the Chinese Clinical Trial Registry (ChiCTR2000029083; January 13th 2020) (http://www.chictr.org.cn/edit.aspx?pid=48279&htm=4).

### Participants

This prospective study was conducted in Peking University People’s Hospital between January 2020 and August 2020. Adult patients (18–75 years of age) scheduled for MVD surgery for hemifacial spasm were eligible. The diagnosis of hemifacial spasm was established according to medical history, clinical manifestation of involuntary facial movements, and neurological imaging [10]. The main exclusion criteria were (1) body mass index (BMI) at < 18 or > 30 kg/m^2^, (2) preoperative diagnosis of motion sickness or vertigo, (3) ophthalmic diseases (e.g., glaucoma, cataract, eye trauma) or previous eye surgery, (4) uncontrolled hypertension (systolic blood pressure > 180 mmHg and/or diastolic blood pressure > 110 mmHg despite of treatment), uncontrolled diabetes (fasting blood glucose > 10 mmol/l despite of treatment), severe cardio-cerebrovascular disease, and mental diseases, and (5) pre-planned immediate return to the intensive care unit after surgery.

## IOP measurement

IOP was measured using a Pulsair Intellipuff portable noncontact tonometer (Keeler Ltd., Windsor, UK), immediately prior to anesthesia induction and 30 min after the patients gained complete consciousness in a supine position, by an experienced doctor not involved in the study otherwise. Two to three measurements were taken for each eye in each patient, and averaged. IOP reduction was defined as IOP decrease by at least 1 mmHg from the preoperative baseline.

## Anesthesia

Anesthesia was induced with intravenous midazolam (0.03 mg/kg), propofol (1.5–2.5 mg/kg), sufentanil (0.3–0.4 µg/kg), and rocuronium (0.8 mg/kg), and maintained with propofol and remifentanil at the bispectral index score between 45 and 55. PetCO_2_ was maintained at 35–45 mmHg. For PONV prevention, patients received 1 mg droperidol and 40 mg methylprednisolone intravenously after intubation, and 5 mg tropisetron intravenously before skin suture. After the resumption of spontaneous respiration, patients received 1 mg neostigmine, 0.5 mg atropine and 0.5 mg flumazenil intravenously, and were extubated. Oxygen supplementation was conducted at a rate of 2 L/min for 6 h.

## PONV assessment

PONV events were evaluated over consecutive 24 h periods, at 9 am on postoperative day (POD) 1, 2 and 3. Severity of nausea was evaluated using a 0–10 numerical rating scale (NRS), with 10 for unbearable nausea [11]. Vomiting included actual vomiting and retching. Rescue tropisetron (5 mg) was given intravenously when nausea score was ≥ 7, or upon repeated episodes of vomiting. Dizziness was also assessed using an NRS.

## Endpoints

The primary endpoint was the rate of vomiting on POD 1. Secondary endpoints were the rate and severity of nausea and dizziness, and postoperative tropisetron rescue on POD 1–3.

## Sample size calculation and statistical analysis

We conducted a preliminary study in 51 patients. The results showed 55.9% (19/34) rate of vomiting on POD 1 in subjects with IOP reduction vs. 11.8% (2/17) in subjects with no IOP reduction. Assuming 90% power, and *α* at 0.05, a total of 82 subjects were required. Assuming a dropout rate of 20%, 103 subjects would be needed.

Continuous variables are presented as mean ± standard deviation (SD) and analyzed using Student’s *t*-test if distributed normally, and presented as median ( interquartile range) and analyzed using Mann-Whitney U test otherwise. Categorical variables are presented as number and percentage, and analyzed using Chi-square test. Variables with *p* < 0.20 in univariate analysis in comparison between subjects with or without PONV were entered into a multivariable logistic regression analysis to identify the variables associated with PONV. All statistical analyses were conducted using SPSS version 25.0 software (IBM, New York, NY, USA). *p* < 0.05 (2-sided) was considered statistically significant.

## Results

**Fig. 1:**
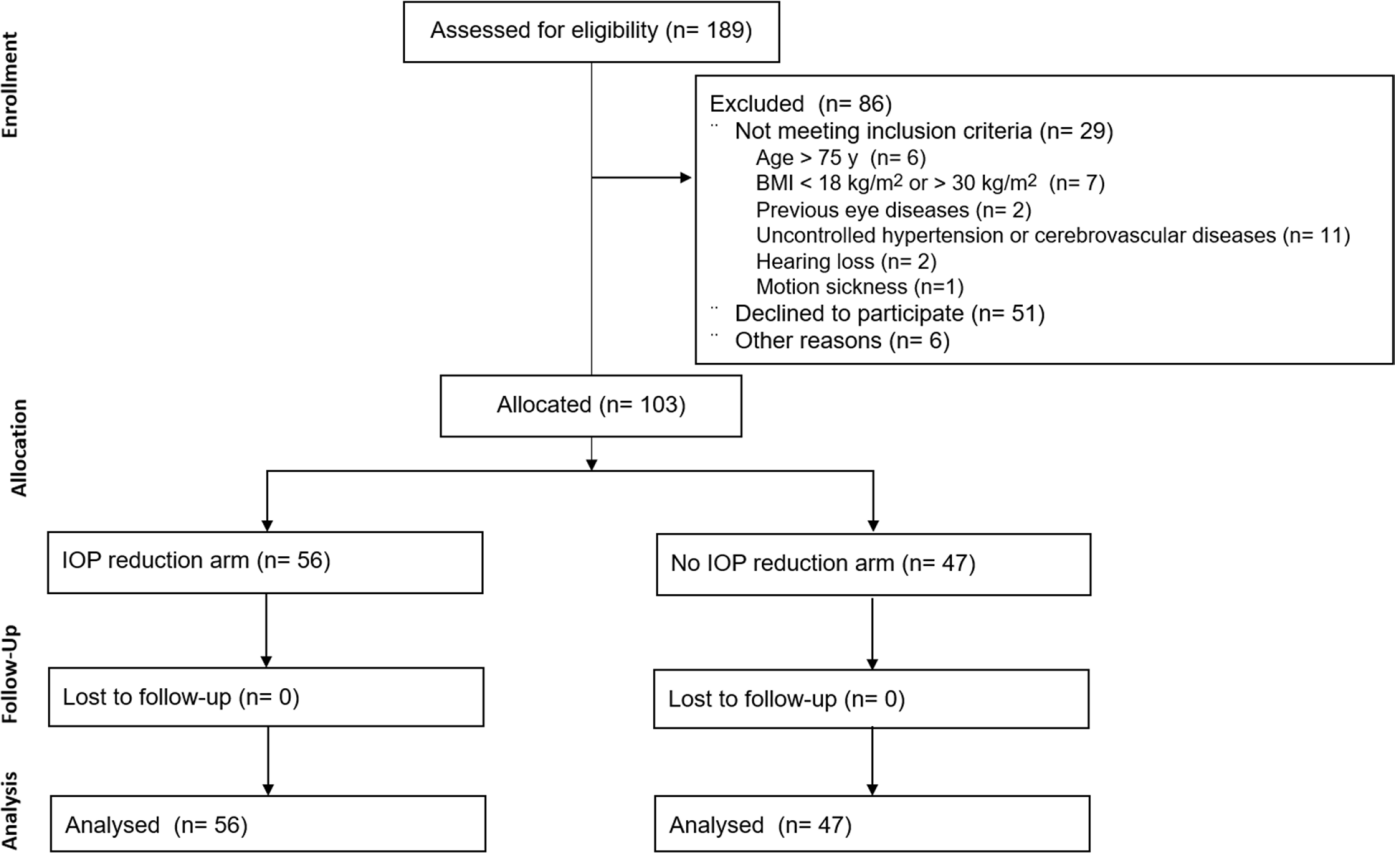
The study flowchart. *IOP* Intraocular pressure, *BMI* Body mass index

A total of 189 patients were screened, and 103 were enrolled (Fig. 1). The final analysis included 103 patients. The mean age was 52.12 ± 9.09 years, with a 1:2 male-female ratio (Table 1). Fifty-six patients (54.4%) had IOP reduction (at least 1 mmHg decrease from the baseline).

**Table 1:** Demographic and baseline characteristics of the study population

	All (*n* = 103)	IOP reduction (*n* = 56)	No IOP reduction (*n* = 47)	*P* value
Age, years, M ± SD	52.12 ± 9.09	51.71 ± 8.85	52.60 ± 9.43	0.626
Female, *N* (%)	70 (68.0)	40 (71.4)	30 (63.8)	0.410
BMI, kg/m^2^, M ± SD	24.55 ± 3.00	24.65 ± 2.67	24.43 ± 3.37	0.179
Smoking, *N* (%)	18 (17.5)	9 (16.1)	9 (19.2)	0.682
Hypertension, *N* (%)	33 (32.0)	17 (30.4)	16 (34.0)	0.690
Hemoglobin, g/L, M ± SD	138.76 ± 15.76^#^	138.82 ± 16.99	138.70 ± 14.30^*^	0.968
Preoperative IOP				
mmHg, M ± SD	16.93 ± 3.11	18.31 ± 3.06	15.30 ± 2.28	< 0.001
>21 mmHg, *N* (%)	15 (14.6)	13 (23.2)	2 (4.3)	0.007

*M ± SD* Mean ± standard deviation, *IOP* Intraocular pressure, *BMI* Body mass index

^*^ *n* = 46, 1 missing. ^#^ *n* = 102, 1 missing

## Changes in IOP

Operative characteristics are shown in Table 2. The preoperative IOP was significantly higher in patients with IOP reduction vs. patients without IOP reduction (*p* < 0.001). Postoperative IOP was significantly lower in patients with IOP reduction vs. patients without IOP reduction (*p* = 0.003).

**Table 2:** Operative characteristics of the study population

	Total (*n* = 103)	IOP reduction (*n* = 56)	No IOP reduction (*n* = 47)	*P* value
Operative time, min, Median (IQR)	59.00 (51.00, 66.00)	58.00 (48.00, 64.00)	60.00 (53.00, 73.00)	0.098
Intraoperative sufentanil dose, mg, Median (IQR)	20.00 (16.00, 25.00)	20.00 (15.25, 25.00)	20.00 (16.00, 25.00)	0.777
Intraoperative fluid, mL, Median (IQR)	800.00 (600.00, 900.00)	750.00 (600.00, 900.00)	800.00 (700.00, 900.00)	0.459
Intraoperative output, mL, Median (IQR)	155.00 (110.00, 310.00)	120.00 (110.00, 220.00)	160.00 (110.00, 320.00)	0.190
Postoperative IOP, mmHg, M ± SD	15.68 ± 2.38	14.99 ± 2.26	16.49 ± 2.28	0.003

*M ± SD* Mean ± standard deviation, *IQR* Interquartile range, *IOP* Intraocular pressure

## Vomiting and tropisetron rescue

**Fig. 2:**
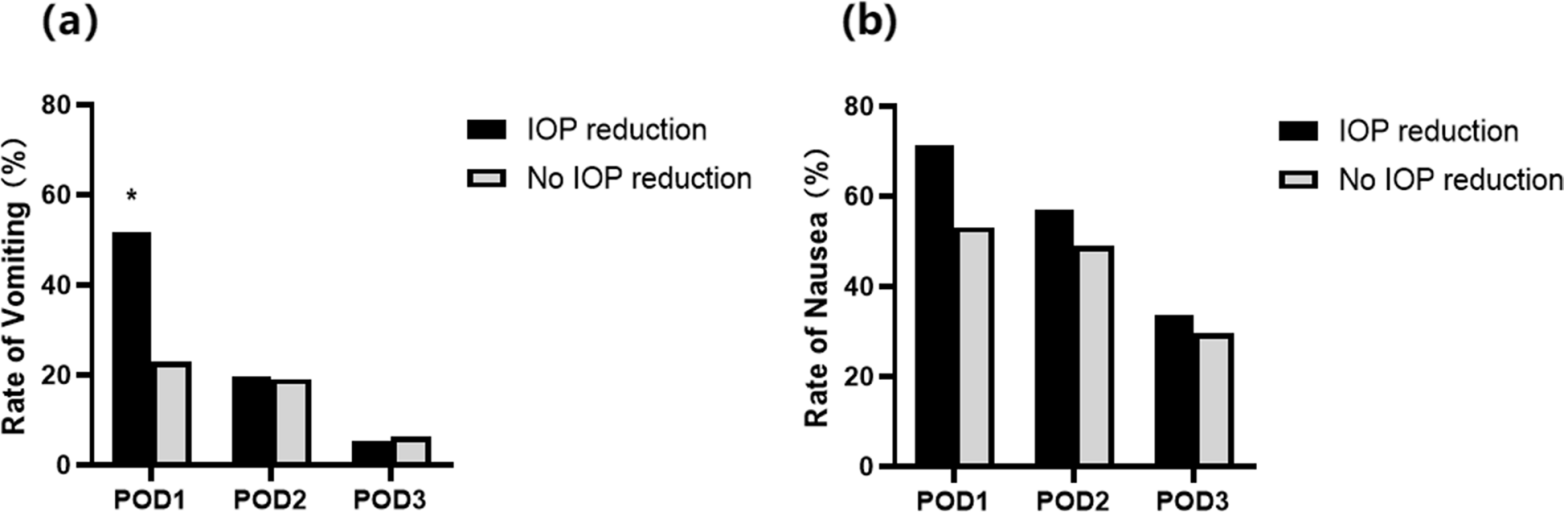
**(a)**: The rate of vomiting on POD 1, 2 and 3. **(b)**: The rate of nausea on POD 1, 2 and 3. Data are analyzed using Chi-square test. *: *p* = 0.003

Forty (38.8%) patients experienced vomiting on POD 1. The rate of vomiting was significantly higher in patients with IOP reduction than those without IOP reduction on POD 1 [51.8% (29/56) vs. 23.4% (11/47), *p* = 0.003], but did not differ on POD 2 [19.6% (11/56) vs. 19.2% (9/47), *p* = 0.950] and POD 3 [5.4% (3/56) vs. 6.4% (3/47), *p* = 0.825] (Fig. 2). Tropisetron rescue during the first 3 postoperative days did not differ between patients with vs. without IOP reduction (21.4% (12/56) vs. 17.0% (8/47), *p* = 0.573).

**Fig. 3:**
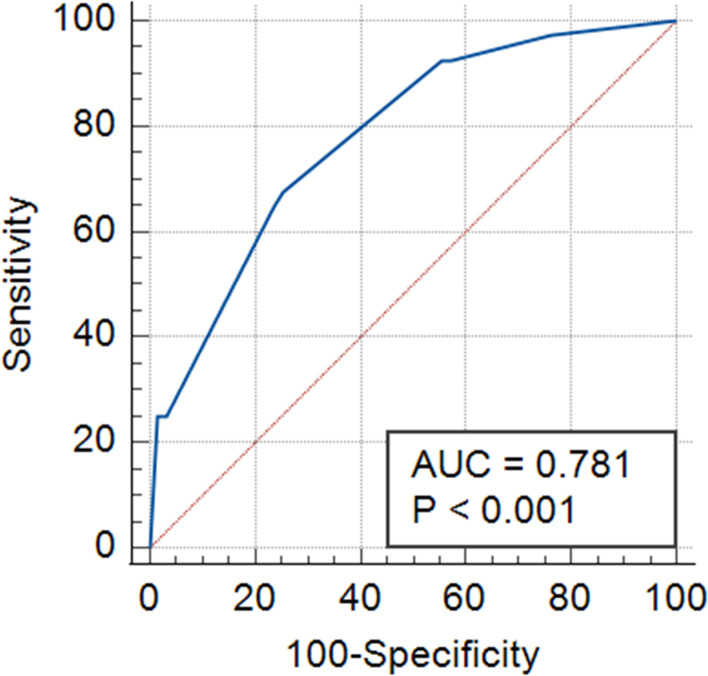
The ROC curve for vomiting on POD 1. The area under the curve on POD 1 is 0.781. ROC, receiving operating characteristics; POD, postoperative day

In the univariate analysis, patients with vomiting on POD 1 had higher female ratio (*p* < 0.001) and higher rate of preoperative IOP > 21 mmHg (*p* = 0.006), IOP reduction (*p* = 0.004), smoking (*p* = 0.017) and higher preoperative IOP (*p* = 0.011). (Additional file 1). In the multivariate regression analysis, IOP reduction and female sex were independent risks of vomiting on POD 1 (Table 3). The area under the curve was 0.781 (Fig. 3).

**Table 3:** Multivariate logistic regression analysis of risks of vomiting on postoperative day 1

	Odds ratio (95% CI)	*P* value
IOP reduction	2.93 (1.13–7.58)	0.027
Preoperative IOP > 21 mmHg	4.05 (0.98–16.69)	0.053
Female sex	7.87 (2.35–26.32)	0.001
Smoking	-	0.555
Preoperative IOP	-	0.707

*95% CI* 95% confidence interval, *IOP* Intraocular pressure

## Nausea

A total of 65 (63.1%), 55 (53.4%) and 33 (32.0%) patients experienced nausea on POD 1, 2 and 3, respectively. No significant difference was observed between patients with IOP reduction and those without IOP reduction [71.4% (40/56) vs. 53.2% (25/47) on POD 1, *p* = 0.056; 57.1% (32/56) vs. 48.9% (23/47) on POD 2, *p* = 0.406; and 33.9% (19/56) vs. 29.8% (14/47) on POD 3, *p* = 0.654] (Fig. 2). The NRS nausea score did not differ between patients with vs. without IOP reduction [2.50 (0, 4.00) vs. 1.00 (0, 3.00) on POD 1, *p* = 0.087; 1.00 (0, 2.00) vs. 0 (0, 2.00) on POD 2, *p* = 0.409; and 0 (0, 1.00) vs. 0 (0, 1.00) on POD 3, *p* = 0.453].

## Discussion

In the current study, approximately 40.0% patients experienced vomiting and 60.0% patients experienced nausea on POD 1 after MVD surgery, despite pre-emptive treatments to prevent PONV. This PONV rate observed in our study is similar to previous studies [3–5]. We also found that IOP reduction after MVD surgery is an independent risk factor for vomiting on POD 1. To our knowledge, this is the first study observing the relationship between IOP reduction and PONV.

From an anatomic point of view, the optic nerve is surrounded by the optic nerve sheath, which is continuous with the dura mater, arachnoid membrane, and pia mater [12], the CSF surrounds the optic nerve sheath up to the point where the optic nerve enters the orbit [13]. Though the intraocular space does not exchange fluid with the retrobulbar subarachnoid space significantly, the IOP can be influenced through the deformation of the lamina cribrosa (a barrier between the intraocular space and the extraocular cerebrospinal fluid space) provided by the pressure difference between these spaces [14]. For IOP measurement, the Goldmann applanation tonometry (GAT) is the gold standard [15], but requires direct contact with cornea. Previous studies showed that, IOP measured with an air puff tonometer agrees well with the results obtained with GAT in both normotensive and hypertensive patients [16–19]. So, we chose a portable noncontact tonometer in our study.

Previous studies have examined the relationship between ICP and IOP. An animal study conducted in dogs showed that, when ICP remained above 70 mmH_2_O, ICP decrease was significantly correlated with a decrease in IOP [20]. Further study conducted in male Sprague-Dawley rats showed that, stimulation of the dorsomedial hypothalamus/perifomical region led to increases in both ICP and IOP, indicating the presence of common regulatory regions of ICP and IOP in the brain [21]. A study of 50 patients showed significant correlation between ICP (as measure with lumbar puncture) and IOP independent of BMI, age and disease type [12]. A meta-analysis that included 546 subjects examined the correlation between ICP and IOP. They found moderate correlation between IOP and ICP, and suggested IOP could be used for intracranial hypertension diagnosis [22]. The included studies in this meta-analysis showed significant heterogeneity, and further studies are needed before using IOP as routine evaluation of intracranial hypertension. The results of our study indicated that, Theoretically, measures that target intracranial hypotension (e.g., prolonged bed rest and fluid infusion) should be considered in patients with robust IOP reduction after MVD surgery. However, multi-centered trials with bigger sample size and more solid study design are needed before translation into clinical practice, and potential risks such as deep venous thrombosis and heart failure should be carefully weighed.

The approximately 40.0% rate of vomiting and 60.0% rate of nausea in the current study was very high, considering the fact that all study subjects received methylprednisolone, droperidol and tropisetron. In contrast, the rate of PONV in western countries is similar despite of less-potent anti-emetic regimen [4]. The reason for such a discrepancy is unknown, but has been previously attributed to ethnicity [23].With regards to the use of tropisetron as rescue treatment, patients undergoing microvascular depression are highly susceptible to severe PONV, and require prophylaxis using multiple antiemetic agents. Option of selecting an agent from a different class is thus limited. A previous study observed significant IOP decrease when using droperidol 5 mg intravenously [24]. In our study, droperidol dosage was considerably lower at 1 mg, with expected less effect on IOP.

Our study has several limitations. First, exclusion of patients with uncontrolled hypertension or diabetes, or severe cardio-cerebrovascular diseases, factors which possibly influence IOP value, could decrease generality of our results. Atropine and neostigmine were given for muscle relaxant antagonism after general anesthesia, which may affect IOP. However, all patients were treated with the same drugs and at identical doses, so the impact was minimal. Not measuring ICP directly represents another inherent weakness.

## Conclusions

In conclusion, PONV occurs in a significant proportion of patients undergoing MVD surgery, and postoperative IOP reduction is an independent and significant predictor of vomiting on POD 1.

## Supplementary information

**Additional file 1: Table 1.** Univariate analysis of risks of vomiting on postoperative day 1.

## Data Availability

The datasets used and/or analysed during the current study are available from the corresponding author on reasonable request.

## Abbreviations

MVD: Microvascular decompression
PONV: Postoperative nausea and vomiting
CSF: Cerebrospinal fluid
ICP: Intracranial pressure
IOP: Intraocular pressure
BMI: Body mass index
NRS: Numerical rating scale
POD: Postoperative day
SD: Standard deviation
IQR: Interquartile range
OR: Odds ratio
CI: Confidence interval
