# Supplementary material for: Association of intraocular pressure and postoperative nausea and vomiting after microvascular decompression - a prospective cohort study
Source: BMC Anesthesiol. 2022 Apr 30;22:132. doi: 10.1186/s12871-022-01665-x (PMC9055703; doi:10.1186/s12871-022-01665-x)
Supplement: Supplementary file 1 — Additional file 1: Table 1. Univariate analysis of risks of vomiting on postoperative day 1. [file 12871_2022_1665_MOESM1_ESM.docx]

**Additional file 1**

**Table 1** Univariate analysis of risks of vomiting on postoperative day 1^*^

|  | No vomiting | Vomiting | *P* value |
| --- | --- | --- | --- |
| N (%) | 63 (61.2) | 40 (38.8) |  |
| Age, years, M ± SD | 51.41 ± 8.68 | 53.23 ± 9.70 | 0.324 |
| Female, N (%) | 34 (54.0) | 36 (90.0) | < 0.001^*^ |
| Body mass index, kg/m^2^, Median (IQR) | 24.51 (22.84, 26.90) | 23.59 (22.55, 27.05) | 0.908 |
| IOP reduction, N (%) | 27 (42.9) | 29 (72.5) | 0.004^*^ |
| Preoperative IOP, mmHg, M ± SD | 16.22 ± 2.54 | 18.06 ± 3.59 | 0.011^*^ |
| Preoperative IOP > 21 mmHg, N (%) | 4 (6.4) | 11 (27.5) | 0.006^*^ |
| Smoking, N (%) | 16 (25.4) | 2 (5.0) | 0.017^*^ |
| Input-output, mL, M ± SD | 583.17 ± 244.44 | 528.8 ± 222.95 | 0.257 |
| Operative time, min, median (IQR) | 60.00 (53.00, 65.00) | 57.00 (47.25, 67.75) | 0.376 |
| Intraoperative sufentanil dose, μg, median (IQR) | 20.00 (16.00, 25.00) | 20.00 (14.25, 25.00) | 0.949 |
| Hypertension, N (%) | 19 (30.2) | 14 (35.0) | 0.608 |

*M ± SD* Mean ± standard deviation, *IQR* Interquartile range, *IOP* Intraocular pressure

*Variables with *p* < 0.20 were included in the multivariate logistic regression model.
